# Scaling digital solutions for wicked problems: Ecosystem versatility

**DOI:** 10.1057/s41267-022-00526-6

**Published:** 2022-06-07

**Authors:** Katherine Tatarinov, Tina C. Ambos, Feichin Ted Tschang

**Affiliations:** 1 Geneva School of Economics and Management (GSEM), University of Geneva, 40 Blvd. du Pont-d’Arve, CH-1211, 3233 Geneva Bureau, Switzerland; 2 Singapore Management University, 81 Victoria St, Singapore, 188065 Singapore

**Keywords:** digital solutions, scaling, ecosystems, technology, wicked problems, United Nations, qualitative, case study

## Abstract

Digital solutions are increasingly used to address “wicked problems” that are locally embedded but require global approaches. Scaling these solutions internationally is imperative for their success, but to date we know little about this process. Using a qualitative case study methodology, our paper analyzes how four digital solutions driven by the United Nations are built and how they scale internationally. These solutions address wicked problems through artificial intelligence, blockchain, and geospatial mapping, and are embedded in networks of partners which evolve during scaling to create unique ecosystem roles and configurations. We identify different ecosystem roles and find that the specific properties of digital solutions – modularity, generativity and affordances – enable either adaptation or replication during scaling. Building on these insights, we derive a typology of four different types of international scaling, which vary in their ecosystem versatility (how the ecosystem changes across locations) and the local adaptation of the application (the problems the solution addresses). This study presents a new way to examine the replication and adaptation dilemma for ecosystems and extends internationalization theory to the digital world.

## Résumé

Les solutions numériques sont de plus en plus utilisées pour résoudre les "problèmes vicieux" (*wicked problems*) qui sont localement encastrés mais nécessitent des approches globales. La mise à l'échelle internationale de ces solutions est essentielle pour leur succès, mais, à ce jour, nous savons peu de choses sur ce processus. À l'aide d'une méthodologie qualitative fondée sur l’étude de cas, notre article analyse les façons dont se construisent et se mettent à l'échelle internationale quatre solutions numériques pilotées par l’Organisation des Nations unies. Ces solutions résolvent les problèmes vicieux par les biais d’intelligence artificielle, de blockchain et de cartographie géospatiale, et sont encastrées dans des réseaux de partenaires qui évoluent pendant la mise à l'échelle pour créer des rôles et des configurations uniques de l'écosystème. Nous identifions différents rôles de l'écosystème, et observons que les propriétés spécifiques des solutions numériques – modularité, générativité et affordances – permettent soit l'adaptation soit la réplication pendant la mise à l'échelle. Nous appuyant sur ces observations, nous élaborons une typologie de quatre types différents de mise à l'échelle internationale, lesquels varient en fonction de la versatilité de l'écosystème (comment l'écosystème change d'un endroit à l'autre) et de l'adaptation locale de l'application (les problèmes auxquels la solution répond). Cette recherche présente une nouvelle façon d'examiner le dilemme de la réplication et de l'adaptation des écosystèmes, et étend la théorie de l'internationalisation au monde numérique.

## Resumen

Las soluciones digitales se usan cada vez para abordar “problemas perversos” que están arraigados localmente, pero que requieren enfoques globales. Escalar estas soluciones internacionalmente es imperativo para su éxito, pero hasta la fecha sabemos muy poco sobre este proceso. Usando metodología de estudio de caso, nuestro manuscrito analiza como se construyen cuatro soluciones digitales impulsadas por Naciones Unidas y cómo se escalan internacionalmente. Estas soluciones abordan problemas perversos mediante inteligencia artificial, cadena de bloques (blockchain) y mapeo geoespacial, y están integradas en las redes de los socios que evolucionan durante el escalamiento para crear roles y configuraciones únicas en el ecosistema. Identificamos diferentes roles en el ecosistema y encontramos que las propiedades especificas de las soluciones digitales -modularidad, generatividad y asequibilidad- permiten la adaptación o la replicación durante el escalamiento. Sobre la base de estos aportes, derivamos una tipología de cuatro tipos diferentes de escalamiento internacional las cuales varían en versatilidad en el ecosistema (cómo cambia el ecosistema a través de los lugares) y la adaptación local para la aplicación (los problemas que la solución aborda). Este estudio presenta una nueva manera de examinar el dilema de la replicación y la adaptación para ecosistemas y extiende la teoría de internacionalización al mundo digital.

## Resumo

Soluções digitais são cada vez mais usadas para lidar com “problemas perversos” que são incorporados localmente, mas exigem abordagens globais. Escalar tais soluções internacionalmente é imperativo para o seu sucesso, mas até hoje sabemos pouco sobre esse processo. Usando uma metodologia de estudo de caso qualitativo, nosso artigo analisa como quatro soluções digitais impulsionadas pelas Nações Unidas são construídas e como atingem escala internacional. Essas soluções abordam problemas perversos por meio de inteligência artificial, tecnologia blockchain e mapeamento geoespacial e são incorporadas em redes de parceiros que evoluem durante a escalada para criar papeis e configurações de ecossistema únicos. Identificamos diferentes papeis de ecossistema e descobrimos que as propriedades específicas das soluções digitais – modularidade, generatividade e utilidades – permitem adaptação ou replicação durante a escalada. Com base nesses insights, derivamos uma tipologia de quatro tipos diferentes de escalada internacional que variam em sua versatilidade de ecossistema (como o ecossistema muda entre locais) e na adaptação local da aplicação (os problemas que a solução aborda). Este estudo apresenta uma nova maneira de examinar o dilema de replicação e adaptação para ecossistemas e estende a teoria da internacionalização ao mundo digital.

## Abstract (zh)

数字解决方案越来越多地用于解决本地嵌入但需要全球方法的“棘手问题”。在国际范围内扩展这些解决方案对于它们的成功至关重要, 但迄今为止, 我们对这一过程知之甚少。我们的论文使用定性案例研究方法, 分析了联合国推动的四种数字解决方案是如何构建的, 以及它们如何在国际上扩展。这些解决方案通过人工智能、区块链和地理空间映射解决了棘手的问题, 并嵌入到在扩展过程中不断发展的合作伙伴网络中, 以创建独特的生态系统角色和配置。我们确定了不同的生态系统角色, 并发现了数字解决方案的特定属性 –  – 模块化、生成性和可供性 –  – 能够在扩展中得以适应或复制。在这些见解的基础上, 我们得出了四种不同类型的国际扩展分类, 它们的生态系统多功能性 (生态系统如何跨地点变化) 和应用程序的本地适应 (方案所解决的问题) 各不相同。本研究提出了一种新方法来检验生态系统的复制和适应困境, 并将国际化理论拓展到数字世界。

*“Digital technology is shaping history. But there is also the sense that it is running away with us. Where will it take us? The answers to these questions depend on our ability to work together across disciplines and actors, across nations and political divides.”*

*–Antonio Guterres,* *United Nations Secretary General*

## INTRODUCTION

Prior IB literature has demonstrated that products tend to be either replicated or locally adapted as they scale internationally (Jonsson & Foss, 2011; Szulanski & Jensen, 2006; Venaik, Midgley, & Devinney, 2004). Recent efforts have been made to extend internationalization theory to the digital world (Autio, Mudambi, & Yoo, 2021; Banalieva & Dhanaraj, 2019; Coviello, Kano, & Liesch, 2017), but do not sufficiently account for the specific properties of digital solutions in their scaling. Digital solutions incorporate data-driven technologies, algorithms, and software to solve problems in new ways and engender closer interaction with the end-user through modularity and affordances (Nambisan, Zahra, & Luo, 2019). Many digital technologies and digitally enabled enterprises not only depend on scale for their economic viability but are themselves based on network effects (Oborn, Barrett, Orlikowski, & Kim, 2019) which can impact their application and scaling. Digital solutions often have large data requirements (Brynjolfsson & McAfee, 2014), so scaling becomes imperative (Bharadwaj, El Sawy, Pavlou, & Venkatraman, 2013; Monaghan, Tippmann, & Coviello, 2020), and requires embedding solutions across new configurations of actors or ecosystems (Li, Chen, Yi, Mao, & Liao, 2019). These unique characteristics of digital solutions call for a different perspective when examining their international scaling.

Recent developments related to the internationalization of multinational corporations (MNCs) have shown the importance of business ecosystems (Ganco, Kapoor, & Lee, 2020; Parente, Rong, Geleilate, & Misati, 2019). These are networks of organizations and individuals that co-evolve capabilities and align their investments to create additional value, improve the efficiency of operations, and innovate in partnerships (Adner, 2017; Iansiti & Levien, 2004; Moore, 1993; Williamson & De Meyer, 2012). Yet, we know little about how digital solutions enable the creation and adaptation of ecosystems when scaling across different locations (Reuber, Tippmann, & Monaghan, 2021). The concept of ecosystems in the IB literature has mostly focused on anchor firms with internal technological capabilities, and on such firms’ relationships with host market firms or other profit-motivated actors (Parente et al., 2019). The related digital solutions are often characterized as ‘distributed innovations’ (Oborn et al., 2019). Many digital solutions address complex problems that are locally embedded but need global solutions and increasingly involve governments and not-for-profit organizations. Such varied ecosystem configurations require an explicit definition of the actors’ roles and contributions.

This challenge of ecosystem configuration and international scaling is amplified when digital solutions seek to address wicked problems (George, Howard-Grenville, Joshi, & Tihanyi, 2016; Rittel & Webber, 1973), defined as complex global challenges that typically require interactions between global actors and local actors in developing regions (Busch & Barkema, 2021; Oborn et al., 2019; Seelos & Mair, 2013), and where cross-sector actors meet with different motivations and behaviors (Buckley, Doh, & Benischke, 2017). Technological solutions are often praised as panaceas that can address these complex problems, but there is a long-standing legacy of failures, often due to developers or donors not accounting for local conditions or users (Chliova & Ringov, 2017; Haug, 1991). In such contexts, the questions of how to configure ecosystems and scale digital solutions internationally are pressing, but are still largely unaddressed in the literature (Kistruck, Beamish, Qureshi, & Sutter, 2013; Seelos & Mair, 2007). To investigate the phenomenon of scaling digital solutions in this context, our research asks: *How are digital solutions for wicked problems scaled across locations, and how are their ecosystems (re-)configured to facilitate scaling?*

We address this question through an inductive case study approach by investigating four digital solutions that aim to tackle wicked problems (Churchman, 1967; Rittel & Webber, 1973). These solutions are driven by United Nations (UN) organizations and address complex challenges that are locally embedded but that require global approaches. They use specific digital technologies – artificial intelligence, blockchain, or geospatial mapping – which are scaled to multiple countries and are distributed by nature (Oborn et al., 2019). Therefore, and in line with recent IB research (Li et al., 2019; Parente et al., 2019), we view the configurations of digital solutions through an ecosystem lens and delve into the actual configurations and re-configurations of each solution’s ecosystem to capture the complexity of the specific context (Brusoni & Prencipe, 2013). By mapping the ecosystem actors in each new location as the digital solution scales, we provide fine-grained detail on the roles of the actors and on the nature of their changes during the scaling process.

This research makes the following contributions to the literature on how digital solutions scale internationally. The first contribution is an identification of the roles different actors play in ecosystems during the internationalization of digital solutions, advancing the scarce literature on how digital solutions scale to different contexts (Li et al., 2019; Nambisan et al., 2019). Second, we develop a typology of international scaling which proposes how the specific properties of digital solutions relate to their replication and adaptation. We derive four types of scaling which vary in their ecosystem versatility (i.e., how the ecosystem changes across locations) and the local adaptation of the application (i.e., the problems the solution addresses). These findings show that combining properties of the digital solution with the ecosystem configuration is key to understanding how digital solutions scale internationally*.* Overall, our study adds to the stream of research that extends internationalization theory to the digital world (Banalieva & Dhanaraj, 2019; Coviello et al., 2017; Strange & Humphrey, 2019) and suggests that the classic replication-adaptation dilemma may be overcome by digital solutions.

## THEORETICAL BACKGROUND

### Unique Properties of Digital Solutions

The role of digitalization on the management of organizations is subject to a lively debate in management research (Davenport, Barth, & Bean, 2012; George, Haas, & Pentland, 2014; McAfee, Brynjolfsson, & Davenport, 2012). Digital solutions are outputs of digitalization as they rely on digital technology, that is any device or service based on algorithms usually constituted in binary computing-based software (Hadlington & Scase, 2018). In addition, digital solutions are innovations suited to solve complex problems in a different way, e.g., as digital services (examples in medicine: Nekorachko, Pkhakadze, & Vlasenko, 2019; deforestation: Watanabe, Naveed, & Neittaanmäki, 2018). There are several key characteristics of digital solutions that are relevant (see for instance Majchrzak, Markus, & Wareham, 2016; Nambisan et al., 2019); of which three properties stand out and differentiate digital solutions from classic technology products: modularity, generativity, and affordances. These are defined generally but with varying aspects.

The first property is digital technologies’ modularity, which is related to the layered technological architecture of the solution (Yoo, Henfridsson, & Lyytinen, 2010). Technical modularity was a long-held concept in engineered systems, repurposed by the innovation management literature to describe how base components can be recombined into different configurations of systems or architectures (Baldwin & Clark, 2000). Digital technologies’ modularity is facilitated by software’s properties. The layered characteristics of digital solutions allow virtual components to be transformed in more ways than physical products, as the layers facilitate additional means of recombining functionalities. Modules as well as information are made transferable by the creation of standardized interfaces between the layers, further facilitating recombination to create new applications or adapt to new user requirements and environments with minimal reprogramming (Baldwin & Clark, 2000; Yoo et al., 2010).

The second property of digital technologies is their generativity. The ‘generative capacity’ of digital technologies was first specified by Zittrain (2006) for the Internet, describing the digital technology underlying the Internet as empowering varied participants to not only build and distribute code and content (Zittrain, 2006) but to also transform and reuse data as part of additional functionalities – all without any hierarchical coordination at the highest levels of the Internet. It “*increases with the ability of users to generate new, valuable uses that are easy to distribute and are in turn sources of further innovation*” (Autio & Thomas, 2020; Zittrain, 2006: 1982). Thus, generativity can also relate to the data-oriented purposes of digital solutions. Consequently, the success of digital solutions depends on the ability to scale and reconfigure them, and on their continuing and iterative advancement (Ansari, Garud, & Kumaraswamy, 2016; Nambisan, Lyytinen, Majchrzak, & Song, 2017).

The third property, affordances, is related to the users’ interaction with the digital solution. These are the ‘action possibilities’ (Nambisan et al., 2019) provided by a digital solution to users and the situations they face. Compared to other technology products, digital solutions are characterized by the opportunity for end-users to have continuous interactions with the technologies and to engage in the co-creation of value with developers and other actors (Iansiti & Lakhani, 2014). Research on affordances shows how different uses and local contexts vary considerably with the same technology (Nambisan et al., 2019). Through a technology’s affordances, the actions that individuals or organizations may take with that technology can shape it to an (often local) purpose or context (Majchrzak & Markus, 2013), and open the door for complementary knowledge partners to participate (Baldwin & Woodard, 2009). This allows the creation of partnerships with more varied meanings (Yoo et al., 2010), and where partners may adopt different roles. The implications of these properties of digital solutions vis-à-vis technology products’ properties are shown in Table 1.

**Table 1:** Differentiating digital solutions

	Technology product	Digital solutions
Technological architecture and capability of base technologies	Often standardized, as lower configurability and physical nature requires growth by replication (e.g., manufacturing processes fixed by design) Subject to economies of scale in production of physical artifacts Specific product components or features may be locally adapted	Layered Modular Architecture and Modularity of Virtual Components: Digital technologies are free of constraints faced by physical products due to their interoperable layers, and thus have greater recombinative possibilities
Distributed nature of innovation and intensity of data	Product development lifecycle is determined by requirements (and completed to fixed schedule). Often structured product development process, tools and product forms are not integrated through a shared digital platform, and product release does not involve continual updating	Generativity: Innovation is distributed due to digital platforms’ capacity to produce unprompted change (driven by large, varied, and uncoordinated audiences self-organizing on platforms) Data can be transformed and reused as part of other digitally enabled functions (e.g., using analytics in/on various outputs).
End-user involvement in innovation	Limited to traditional end-user role as user of ‘product as designed’ User-led innovation can still be invoked for products as means for extending products (though not for extending products’ functionalities as much as digital)	Affordances: User-contexts reflecting differences in institutional environment may shape technology use and lead to participatory innovations (involving users) Co-creation enables new functionalities with end-users driven by specific user needs

The three properties have two important implications for how digital solutions emerge and grow. First, digital solutions require close interaction with end-users, but also with partners across different sectors, which means that digital solutions are often embedded in ecosystems of different stakeholders (Autio & Thomas, 2014; Li et al., 2019). These ecosystems are necessary for facilitating the co-creation and iterative data feedback loops. Second, most digital technologies and digitally enabled enterprises not only depend on scale for their economic viability but are themselves based on network effects (Oborn et al., 2019). At the same time, they involve large amounts of data and may require reconfiguration, which further adds to their distributed character. This means that scaling, and an understanding of that scaling process, becomes important (Bharadwaj et al., 2013; Monaghan et al., 2020). However, we still lack insights into how these properties affect the interactions of different players in the ecosystem as well as the patterns of international scaling (Li et al., 2019).

### Scaling Digital Solutions Internationally

Scaling is a critical issue in international business and even more so when digital solutions are concerned (Monaghan et al., 2020). Scaling has been described from the perspective of the international expansion of a product or service in the MNC (Jonsson & Foss, 2011; Szulanski & Jensen, 2006) as well as from that of the growth of entrepreneurial firms (Coviello et al., 2017; Monaghan et al., 2020). In the first perspective, scaling largely involves replication across country borders as it relates to specific knowledge (Kostova & Roth, 2002), international entry (Tallman, Luo, & Buckley, 2018; Vermeulen & Barkema, 2002), entrepreneurial growth (Gulati & DeSantola, 2016; Hennart, 2019; Monaghan & Tippmann, 2018), and more recently, industry relationships (Monaghan et al., 2020). In parallel to the prominent integration-responsiveness dilemma (Doz & Prahalad, 1991), a “replication dilemma” often emerges between desiring the benefits of replicating a model exactly and needing to adapt it to the salient characteristics of new international environments (Devinney, Midgley, & Venaik, 2000; Winter & Szulanski, 2001). The ability to scale through replication in a new environment may also be severely constrained by forces beyond the recipients’ control, such as government regulation (Teece, 1998), incompatible technologies (Kogut & Zander, 1992), or inadequate resources (Pfeffer & Salancik, 1978).

The second perspective is on the growth of digital entrepreneurial firms (Coviello et al., 2017; Monaghan et al., 2020) which refers to high-growth firms as ‘scale-ups’ (DeSantola & Gulati, 2017; Duruflé, Hellmann, & Wilson, 2017; Gulati & DeSantola, 2016) and sheds light on the role of digitalization in their internationalization. However, prior literature on scaling new technology (Autio, Nambisan, Thomas, & Wright, 2018; Hennart, 2019; Monaghan et al., 2020) does not sufficiently discuss the particular variety of international scaling (Nambisan et al., 2019) that we address in our paper – the scaling of specific digital solutions (rather than products or firms).

Digital solutions have been held as a panacea for solving many global challenges, from communications and new ways of working to solving market imperfections and empowering the poor (Forti, 2018; Gray, 2016; Millard & Carpenter, 2014; Quibria, Tschang, & Reyes-Macasaquit, 2002). When digital solutions addressing wicked problems are scaled, there is additional complexity from the newness of the technology and the need to adapt it to emerging demands that are often deeply embedded in the cultural and institutional environment of developing economies (Ambos & Tatarinov, 2022). Such complexity includes the magnitude of the problem, the (lack of) formal institutions, and the specific power constellations of governmental and non-governmental stakeholders, as well as political risk and corrupt environments that often characterize disadvantaged communities (Sartor & Beamish, 2014; Sun, Doh, Rajwani, & Siegel, 2021; Vaaler, 2008). The notion of replication via templates has been proposed for the development context (Chliova & Ringov, 2017), but as we show, scaling across rugged developmental contexts can challenge these conceptions. As the scaling of digital solutions increasingly involves the use, transformation and new combination of sophisticated technology, the configuration and activities of the supporting partners can be expected to be similarly transformed. Even as local knowledge becomes a key resource, the rise of more sophisticated users with the capability to co-create additional services may alter digital solutions’ trajectories. This demonstrates the technology’s affordances (Oborn et al., 2019), some of which can only be activated through scale (Bharadwaj et al., 2013; Monaghan et al., 2020). It can thus be surmised that the *“reproducing (of) a productive system of practices in multiple locations”* may not succeed without some adaptation (Ringov, Liu, Jensen, & Szulanski, 2016: 3; Winter & Szulanski, 2001; Winter, Szulanski, Ringov, & Jensen, 2012). In sum, the mechanisms of international scaling are not clearly understood, and we know little about the implications of scaling digital solutions.

### An Ecosystem Perspective

Since ecosystems have become an important avenue for the creation of digital solutions, the role of ecosystems can be predicted to become even more important when scaling across countries. Digital solutions are likely to be created out of latent capabilities embedded in ecosystems and partners and when internationalizing, ecosystems often need to be reconfigured rather than simply replicated (Li et al., 2019; Nambisan et al., 2019). As firms adapt to changing conditions, business models can usually be reconfigured (Massa, Tucci, & Afuah, 2017). But in situations with multiple partners, reconfiguration of the ecosystem may be another mechanism that occurs during the scaling process (Autio & Thomas, 2014). This also happens to be an important gap in research. Namely, ascertaining the ecosystems’ role in helping organizations create sustainable value through growth.

The strategy literature has examined the ecosystems involved in MNCs’ scaling to new countries (Parente et al., 2019) and there is a nascent literature on platform governance design for digital ecosystems (Chen, Yi, Li, & Tong, 2022; Zhang, Li, & Tong, 2020). While such studies show that ecosystems are important for digital solutions and that challenges arise when internationalizing strategies cross over to weak institutional environments, we know comparatively little about how the configurations of ecosystems may change during the scaling of a digital solution. Understanding the configuration of ecosystems matters since potential partners and realizable ecosystems may differ or be unavailable across regional boundaries and for certain contexts.

Our study focuses on how lead organizations adapt core technology embedded in a reconfigurable application across different international locations. The broader innovation ecosystem literature from the strategy perspective recognizes that the governance of innovations in ecosystems involves informal mechanisms by lead actors as well as control over the product or other architecture (Chen et al., 2022). This perspective highlights the role of the lead firms – stressing a gap in the information systems literature which *“predominantly considers ecosystems in the context of software artefacts and platforms, the architecture and functionalities of which allow multiple constituents to connect and interact”* (Autio & Thomas, 2020: 21). The emerging IB literature on digital ecosystems (Li et al., 2019), where we make our contribution, recognizes these gaps between the information systems and business innovation ecosystem literatures, especially regarding digital solutions being reapplied to different locations, but it does not sufficiently address the issue of how diverse participants in local ecosystems may interact with global partners and how features of the local ecosystems dictate the adaptation process.

Based on the above literature review, we identify a pertinent research gap on how digital solutions scale internationally, particularly when they address complex problems that are locally embedded but that need solutions of a global character; and that involve digital properties which affect their ecosystems’ (re)configuration.

## METHODOLOGY

### Research Context: Digital Solutions for Wicked Problems

Many development problems can be labelled “wicked problems” with complex properties that make them difficult to define and impossible to find optimal solutions for (Churchman, 1967; Rittel & Webber, 1973). Such problems are deeply locally embedded but at the same time globally intertwined (Ambos & Tatarinov, 2022). This applies to poverty, climate change, or migration (Churchman, 1967; George et al., 2016; Rittel & Webber, 1973), where the root causes may be multiple and entangled, and where behaviors and practices are socially and culturally embedded (Collier, 2007). Due to the public good characteristics of such problems, in the absence of strong governmental capabilities, collaborative partnerships have become an increasingly visible way of addressing them (Prahalad, 2005). Given its status as a major global actor tasked with addressing crises in challenging development contexts, the UN has been at the forefront of developing solutions to these problems.

To capture the salient features of this context, our research explores the ecosystems of digital solutions originating from four prominent UN agencies and targeting wicked problems. As multilateral, inter-governmental organizations, the missions of these organizations are to provide solutions for local challenges, but also to scale these for implementation globally (Ambos & Tatarinov, 2022). While the UN is facing increasing pressure to foster a greater impact through innovation driven by digital technology and to do so at a global scale (Voegtlin & Scherer, 2017), digital solutions require organizational resources and processes to develop (Burgelman, Maidique, & Wheelwright, 1996). Because the UN rarely has the resources and internal expertise to develop digital solutions in-house, such solutions are embedded in a network of multiple partnerships – across multiple phases ranging from development to iteration and scaling. To understand how different players are brought together in ecosystems for the global scaling of digital technology, this context provides a well-matched setting for our research. Following the approach taken by prior studies exploring replication (Chliova & Ringov, 2017; Winter & Szulanski, 2001), we seek to identify mechanisms that can better specify the “who”, “what”, and “how” of digital solution’s ecosystems as they scale internationally.

### Research Design and Sample

To address our research question, we opted for an inductive research design to capture emergent insights as a basis for theory building from salient case studies (Miles & Huberman, 1994; Yin, 1994). Case study research is particularly well-suited to understanding such complex phenomena, examining processes, and explaining how certain actions lead to specific outcomes (Edmondson & McManus, 2007; Eisenhardt, Graebner, & Sonenshein, 2016; George & Bennett, 2005). The qualitative approach has been widely used in IB research, particularly when looking to understand a novel phenomenon in an understudied empirical context (Awate, Larsen, & Mudambi, 2015).

Based on a snowball sampling technique (Noy, 2008), we mapped all UN digital solutions that we could identify in 2017. This approach was necessary due to the rarity of digital solutions in these organizations at the time. The team managed to negotiate access to the four solutions which met our sampling criteria: to be based on frontier technology, beyond the proof-of-concept phase, and scaled to at least two locations. Two solutions were aimed at the individual as the end-user and two at institutional end-users, such as governments. This distinction was important because it enabled us to examine the relevance of end-user type when researching the evolution of the initiative as it scaled.

### Data Collection

The research team gained preferential access to key decision-makers in the HQ (New York, Rome, Geneva) of these organizations as well as several initiative implementors working in the field offices, such as in Pakistan and Kenya. We built a large database of primary and secondary data relating to the development of the focal digital solutions. The primary data collection included 24 interviews with stakeholders to understand how these solutions scale and their ecosystem in each location. These interviews were semi-structured to allow for deep inquiry (Rubin & Rubin, 2011) and conducted with individuals who were directly involved in the initiative. In addition, we hosted several workshops to bring together innovation leads from these organizations to discuss their experiences. Additional secondary data sources were consulted and added during the research process, such as organizations’, governments’, and partners’ annual reports; press releases; consultants’ reports; and UN internal publications.

During the 36 months of the data collection process, and as themes emerged, the authors went back to multiple people involved in the solutions to check on the development of the project and clarify open questions until saturation was reached. Despite following the solutions for 3 years, we had to draw on some retrospective accounts. By relying on multiple informants, several of whom covered the entire evolution of the initiative, we reduced potential retrospective biases (Golden, 1992; Jick, 1979; Leonard-Barton, 1990). Table 2 summarizes the data sources and characteristics of the four case studies.

**Table 2:** Solutions’ characteristics and data collection

Solution characteristics	Alcott	Butler	Carter	Desai
Description	Blockchain technology to deliver cash payments in refugee camp	Citizen & youth engagement platform	Predictive analysis AI platform to forecast population movements	Geospatial data for population mapping
Primary end-user	Individual	Individual	Institutional	Institutional
Launch year	2017	2011	2016	2017
Org/HQ location	Org A/Rome	Org B/New York	Org C/Geneva	Org D/New York
Locations	Pakistan; Jordan; Bangladesh	Global – 68 countries	Somalia	Nigeria, DRC, Zambia, Mozambique, S Sudan, (10 countries total)
Current situation	Scaling	Developing new services and building on existing tool	Being spun-off	Scaling
Technology	Blockchain	SMS platform; AI chatbot	Artificial Intelligence	Geospatial mapping
Reach	600,000 refugees	10 million users	22 regions mapped	10 countries mapped
Humanitarian impact	Increases ease, ability to pay for food	Empowers communities by giving a voice to youth	Helps governments prepare for refugee crisis situations	Supports informed government decision-making in low- and middle-income countries
Solution purpose	Trust-based transactional assurance	Analytics; Data collection	Predictive analytics	Analytics; Assisted decision-making
Data collection				
Interviews*	7	6	7	4
Observations**	2		1	
Annual reports	6	4	5	6
Press releases	8	25	10	15
Presentations	2	1	2	

^*^With initiative leads, data scientists, or other initiative stakeholders

^**^Observations involved a researcher spending time with the team in their offices as they worked on the initiative

### Data Analysis

Our research followed established procedures and used each case as a stand-alone ‘experiment,’ commencing with a detailed within-case analysis before progressing to the cross-case comparison (Eisenhardt, 1989; Yin, 2015). We began by writing up cases chronologically and used temporal bracketing to analyze the international scaling patterns (Langley, 1999). In line with our theoretical background, we used an ecosystem lens to structure our data collection following the definition of ecosystems as cooperative governance modes requiring value creation by multiple co-specialized partners (Adner, 2017; Autio & Thomas, 2014; Jacobides, Cennamo, & Gawer, 2018; Li et al., 2019).

First, we identified key actors in each ecosystem. After completing this step, we validated findings with each initiative’s lead. This process revealed the need to use a more fine-grained analysis to surface the local–global interactions in the ecosystem. Consequently, we also included the more salient intra-organizational relationships of each case.

In the next step of the analysis, we compared the specific roles that actors took in the ecosystem. We began our initial investigation by using a coarse-grained categorization of three roles – orchestrator, integrator, and complementor (Nambisan et al., 2019) – and further refined these roles through the analysis. The orchestrator is the ecosystem leader which drives the initiative forward and initiates the scaling. Such ‘lead’ or ‘hub’ firms have provided ecosystem stability by ensuring the creation and extraction of value without the benefit of hierarchical authority (Dhanaraj & Parkhe, 2006; Iansiti & Levien, 2004). The integrator is the partner with the deepest contextual knowledge in the initiative; it provides support to the orchestrator and enables the connections to other partners, such as a country office, government partner or strong academic partner (like Celuch, Bourdeau, Khayum, & Townsend, 2017). Given the differing organizational landscapes and usage contexts across country borders, the ‘integrator’ role is defined more loosely here than in other systems integration contexts. It is separated from the orchestrator, whereas in a traditional technology-based ecosystem, the hub firm may fulfill both roles as part of its architecting or blueprinting function. In our context, more knowledge-based layers that involve anything from highly specific user knowledge to statistical knowledge on a range of geographic dimensions may involve integrators to take on a broader range of roles.

The complementors in the ecosystems we studied were also varied, due to the ecosystems’ knowledge-, service- and technology-based nature, so we define complementors to include partners that provide market access to the end-user (i.e., knowledge about them), partners that aid with initiative delivery and visibility, and the technology developers. These actors subsume the activities that will help the initiative expand (Dedehayir, Mäkinen, & Ortt, 2018; Williamson & De Meyer, 2012). We created a graphical representation for each ecosystem map and started with detailed within-case analyses to understand how these ecosystems changed with scaling. Finally, we conducted cross-case analyses, using pattern-matching techniques to compare and contrast the overall patterns of international scaling of the four cases, to surface insights on the different ways in which digital solutions scale and how their ecosystems evolve during this process.

## FINDINGS

The findings section provides a rich description for each of the solutions, including the interplay between the digital solution and its evolving ecosystem. It portrays the challenge the solution was aiming to solve, the scaling process and the ecosystem partnerships established in each location, highlighting the roles of the actors as their solutions expanded globally. The cross-case comparisons and figures at the end of this section provide a summary of the ecosystem actors, the reach (i.e., the number of users or scope of application), and the nature of the technology’s application as the solution was scaled.

### Alcott

#### The challenge

The mission of Org A is to fight hunger worldwide, providing food assistance where it is most urgently needed – during and after conflicts and natural disasters. A major part of this work involves providing aid in the form of cash-based or income transfers (as opposed to aid being tied only to food) to beneficiaries. “*40% of our workload is comprised of cash-based transfers and that part of our business is growing year over year*,” explained the Head of the Change Management Division at Org A. However, the movement of money to 14 million recipients globally incurs high fees, and is marred by financial risk, due to the instability of banking services in these conflict-torn situations. In addition, negotiating transfers through banks requires the client’s personal data to be shared, causing customer privacy to be a major issue. Occasionally, data were lost in transfers involving these and other third parties, due to their opaque processes. In addition, Org A often had to operate multiple platforms and solutions to provide cash to its diverse country and client base, increasing the complexity of earlier solutions.

#### Solution development

In January 2017, an accountant working in an Org A office approached the organization’s Innovation Accelerator, the *Orchestrator*, with an idea to develop a blockchain solution to improve how the organization transferred cash. The ideation process stretched into a bootcamp where the idea was refined by a small team consisting of the accountant, a blockchain expert, and the Accelerator team. After several iterations, the tool was ready to pilot, and a global consultancy firm was brought in to advise on and analyze the potential risks relating to the introduction of the blockchain technology.

#### Ecosystem: Location 1

The digital solution *Alcott* piloted by the *Orchestrator* in Pakistan was designed with the purpose of aiding the transfer of money via blockchain technology. The team hoped to confirm basic assumptions around the blockchain technology’s effectiveness in managing cash transactions (in combination with personal biometric or other signatures unique to individuals). “*We need to transfer money to a lot of [refugees] whose identity we are not entirely sure of at all times. We also need those transactions to be secure and at a lower cost”*, explained an initiative team member. Organizational staff observing these issues in the field recognized the need for a neutral platform that would tackle these challenges. “*It is not about the technology. We know that technology is a way to get greater efficiency and effectiveness, but our end goal is – dignity of people, efficiency and effectiveness. The goal of the blockchain project is to increase efficiency and transparency and accountability,”* the Head of the Change Management Division explained. The cash was transferred through a blockchain-backed system for distributing and recording transactions, integrated with identity-confirming iris-scanning technology which was already being employed in the camp by a partner of the *Integrator*, Org A’s Pakistan office. This integration was possible due to the generative and modular potential of the blockchain solution, which enabled it to be paired with the digital identification solution (and as motivated by the previous work of the local country office with sister agencies on the ground). All refugees had their own unique biometric identification (based on a technology management system developed by another organization). The blockchain solution increased the transparency and traceability of the transfers and facilitated a more empowered existence for the refugees by providing them with digital identities while also removing the middlemen providing insecure transactions.

The ecosystem in Pakistan included diverse actors acting as *Complementors*: a global technology company specializing in blockchain infrastructure and an enterprise blockchain solution company which launched the initiative with the organization and aided with the on-going technology development. The *Orchestrator* (Org A Innovation Accelerator) provided seed funds for the initiative and worked closely with the *Integrator* (Org A Pakistan country office) to ensure the acceptance and adoption of the solution by the recipients in the refugee camps, as well as the onboarding process. A large government aid agency became an additional *Complementor* by providing further funding. The initiative was piloted successfully with 100 people before the *Orchestrator* decided to test it in another location in the same country to confirm that the technology was easily transferrable. Regarding the introduction of these new programs, the head of the Change Management Division explained the challenges of working with diverse actors in the ecosystem:

The first constraint…is understanding what is happening on the ground and […] the operational constraints: do you actually have the ability to mount one of those programs locally? Is the program aligned with what the host government wants to do and their policy of providing assistance? And then, there are different donors that we have that provide us the opportunity to have these programs – they may have different preferences and risk appetite.

The implementation in Pakistan was successful, providing a 98% reduction in local bank fees for Org A, while replicating the ideal beneficiary experience of the function. Thus, by mid-2018, Org A was also exploring how blockchain technology might be used in its other workstreams, such as supply chain operations and digital identity management, which illustrated the potential of the technology and its characteristic of having high 'affordances’. Even with these developments, the team’s more immediate goal was still to scale the tool to other locations.

#### Ecosystem: Location 2

The second location chosen by the *Orchestrator* was a refugee camp in Jordan where the team began implementation to cover 10,500 Syrian refugees. The ecosystem of partners in this new expansion again involved the same technology firm, which continued developing the blockchain solution. In addition, enabled by the modularity inherent in the base technology solution, a sister agency joined the project as a global partner:

…[the two organizations] validate each other’s transaction through the common use of [Alcott], which reduces fragmentation in humanitarian assistance.

Other *Complementors* also continued to adapt the technological solution to the local requirements in Jordan. By 2018, *Alcott* was providing more than USD $1 million worth of cash-based transfers through 100,000 transactions while reducing local banking fees by more than 90%. In 2019, the initiative also started reaching refugees in a second camp in Jordan with 107,000 Syrian refugees.

#### Ecosystem: Location 3 and scaling

After initial pilot and testing in late 2019 and early 2020, the initiative officially launched in the world’s large refugee camp – in Bangladesh – to accelerate the impact of the initiative. As explained in the news brief from the time:

The technology was introduced to bolster the assistance that [Org A] provides people while it faces COVID-19. As of September 2020, the initiative was servicing over 500,000 of the 855,000 Rohingya refugees in Cox’s Bazar, and [Org A] planned to extend its use to all of them before the years’ end.

For Bangladesh, the technology was adapted from the original base identification technology (which used iris scanning) to one using QR codes for identification, allowing refugees to still receive a digital identity (code) that distinguished individuals from each other, without revealing their true identities for security and privacy reasons. This change in identification technology highlights the modular nature of the tool as it adapts to the needs in the existing environment and the technologies there. The reason QR codes were used in Bangladesh was that the refugee camp was not equipped with the iris scanning technology that was available in Jordan’s camps. The academic partner invited to evaluate the future strategy of the project explained the difference in this new location:

In the case of Jordan, the technological infrastructure for Cash Based Transfers exists, which is not the case for Cox’s Bazaar, Bangladesh, where energy access/supply and technical capacity can impede delivery of cash assistance through blockchain. Culturally and in the humanitarian community, new technology can be perceived as risky due to the potential for data mismanagement (Awan & Nunhuck, 2020).

The established partners remained for the new implementation in Bangladesh. The Business Development lead at the *Orchestrator* explained, “*We wanted to scale up to show that this initiative can bring true value to the organization.”*

### Butler

#### The challenge

Org B is a UN agency responsible for providing humanitarian and developmental aid to children worldwide. Youth involvement in policy-setting and developmental decision-making is heavily embedded in local institutions and is thus a wicked problem due to the difficulty of framing and solving the problem, including discovering its root causes. The Uganda country office, the *Orchestrator* for the first location, had identified several youth challenges related to engaging young people’s opinions: such as unemployment; poverty and high school dropout rates; but also noted the potential arising from the increasing penetration rate of mobile phones in the country.

#### Solution development

In May 2011, under the leadership of the *Orchestrator* (Ugandan country office), Org B developed an open-source SMS platform that supported data collection and youth engagement activities. The country office hired a former IT and digital media consultant to build on the platform and create a mobile-based application that could communicate directly with youth. The manager and his small team created the tool now called *Butler*, which aimed to give an opportunity to every young person in Uganda to participate in the decisions that affected them and to take an active role in informing the development of the country – promoting transparency and accountability at the grassroots level.

#### Ecosystem: Location 1

In Uganda, Org B partnered with a global computer hardware company, to develop the tool to reach national scale as a *Complementor*. A news release from the launch explained: “*Since February 2013, [Butler] has been using text analytics and machine learning technologies from [global tech MNC] to help deal with the flood of information by automating the classification of messages*.” This global level partnership was enabled by the inherent modularity of the tool that let other partners build on the existing layers, as well as the affordances that allowed for further co-creation of the solution by local partners together with a large technology company.

Equally important as the technology and its partner to the success of the application was the country office’s partnerships at the local level with the government, NGOs, and youth organizations. Young people were encouraged to join *Butler* through local non-governmental organizations (NGOs), youth groups (such as Scouts, Girls Education Movement), and faith-based organizations – these being *Integrators* that connected with the youth responders (i.e., users). The government also used the data gathered through the tool to help shape policy. Responses received by SMS on *Butler* were analyzed in real time and the data were mapped at the local level and compiled nationally. *Butler* users were made anonymous to protect young people when sharing sensitive information. After the initial pilot in Uganda reached 200,000 people, *Butler* started scaling in neighboring countries, reaching Zambia and Burundi in 2012.

#### Ecosystem: Location 2

The Zambia country office, the *Orchestrator* in location 2, sent an engineer to Uganda to work closely with the *Butler* team in the original location to understand the system and develop the strategy for Zambia. The *Orchestrator,* in partnership with a local health council as the *Integrator*, launched *Butler* in Zambia in 2012. It was built on the foundation of its Uganda counterpart and was further developed through a participatory, consultative process including a design workshop that involved capturing user needs and preferences from its diverse stakeholders. While this process was made possible by the generative properties inherent in digital platforms and other innovations, organizational approaches such as these workshops are needed to bring in the perspectives of potential users (and other stakeholders) in a design-like process, thus bridging the gap between the traditional ‘technology’ development paradigm and the ‘action potential’ inherent in the media technology’s affordances. Local mobile phone operators acted as *Complementors,* providing SMS services to *Butler* at discounted rates*.* The difference in Zambia’s version of the tool was the application’s particular focus on HIV/AIDS. These recombinations were made possible by the platform’s modularity, while the affordances culminated in the development of a dashboard enabling partners to respond to individual messages from any channel. *“One million voices are more powerful than 1000 voices*,” explained one *Butler* Coordination Specialist.

#### Ecosystem: Location 3 and scaling

After 2012, the initiative scaled quickly reaching 11 countries by 2014. As explained by a Coordination Specialist, “*In each country where [Butler] scaled, the tool was deployed for a specific purpose and contextualized to that environment.”* One of the largest implementations was in Nigeria in 2014, where the tool was used to help Org B workers share critical information about diseases such as Ebola and polio, and conditions such as new-born care for families living in remote areas. The initiative was spearheaded by the Org B Nigeria country office, the *Orchestrator* in Location 3, with a national youth group as the *Integrator – *that is, the main local partner and key player in the recruitment of youth responders. The government acted as a *Complementor* as a key supporter of the initiative, with top politicians using *Butler* to connect with their communities on critical issues for ascertaining their needs and desires for improvement in the delivery of public services. The number of users on the platform in Nigeria hit one million in less than a year.

#### Global scale

As the tool scaled, new capabilities were added based on the original technology backbone. “*The software that is the backbone of [Butler] needs to be able to take in the massive scale the tool is seeing. It needs to be able to handle 100 million people easily*” explained the *Butler* lead. In developing countries, SMS remained the most used channel of delivery (65%), but other digital channels (i.e., social media modes) gained traction as *Butler* expanded global partnerships to private sector companies to use communications platforms such as Facebook messenger, Viber, Telegram, LINE and WhatsApp; highlighting the modular nature of the tool. *Butler* also used artificial intelligence on its *Butler* Bots to learn about users and interact with them in private on sensitive issues. The *Butler* Bots were locally adaptable and represented a ‘smart’ approach that could respond to queries via SMS and digital channels. With this ability, *Butler* bots could answer ten times more questions at a much lower cost, as more users became digital and allowed *Butler* to dramatically increase its reach and impact on youth around the world. This (use of AI) is an example of extending the application’s functionality so that it increases the user affordances inherent in the application. The data from *Butler* were used to inform high-level policy decisions both at the country level and at global UN resource allocation meetings.

### Carter

#### The challenge

Org C is a global organization dedicated to saving lives, protecting rights, and building a better future for refugees. In 2017, when Somalia was teetering on the brink of famine, the organization’s Somalian country office feared that it would create a surge of refugees overwhelming the organization’s resources. The Country office approached the Innovation Service in its headquarters, the program’s eventual *Orchestrator*, to ask if it was possible to predict the number of the arrivals to the region. “*That was a challenging question for me,*” the Data Scientist at the Org C Innovation Service said, “*but it was the question that launched [Carter]*.” Understanding and trying to predict human migration patterns aimed to innovatively address the need to help vast numbers of humans facing adverse conditions, a wicked problem (with a difficulty of framing) given how exceedingly ill-defined it is by the many uniquely interacting root causes of each refugee crisis.

#### Solution development

Org C (as *Orchestrator*) had already been experimenting with predictive analytics for two years before the request, and the team had sought out value-based partnerships, including ones with large public sector entities, academia, and other UN institutions. The collaborative partnership model granted them access to data, resources, and expertise that was not available in-house. With the collaboration of these organizations – and with the support of a flagship innovation initiative of the UN’s Secretary-General on big data – the team was able to build an analytics model that used meteorological data to predict the refugee population flow into Greece. The Greece experiment was discontinued in 2016, but the insights and partnerships gained during that project prepared Org C’s Innovation team to respond to the request from the Somalia country office.

#### Ecosystem: Location 1

For the solution developed in Somalia, the *Orchestrator* built partnerships with 14 organizations (i.e., *Complementors*) to source seven years of data on varying dimensions of the problem for the experiment. Using supervised machine learning, Org C then designed *Carter*, a statistical engine that was fed data and used trained models to predict the displacement of people in Somalia. The ambition of this initiative, the project manager explained, was “*to increase the capability of Org C, in this case, to be able to predict and prepare better*.” The Somalia Country Office acted as the *Integrator,* which assisted in sharing the operational context and knowledge of the region, as well as in connecting to other of the organization’s sub-units in Somalia who also shared their data. NGO partners acted as *Complementors* by providing additional data to aid the development of the project*.* Data scientists from a sister UN organization acted as mentors in data science and artificial intelligence, and a global NGO provided technical training. According to the news brief from Org C, “[*These NGOs’] data and technical knowledge on climate, weather and market prices is key for the development of this project. These data represent those influential factors for [human] movement in an operational context where those who are forcibly displaced, highly depend on them.”* By 2018, the initiative was able to predict the displacement of persons in 18 regions in Somalia a month in advance. “*[Carter] has shown us that we can be doing so much more with data, especially when it’s openly shared,*” explained the data scientist.

#### Ecosystem: Location 2

After scaling to twenty-two regions in Somalia the team struggled with scaling the project further. The goal was to make this initiative the backbone for decision-making in the organization. A news brief at the time explained, “*The Innovation Service team hopes that [Carter] will become a standard decision-making tool at Org C*.” But as the team was starting to think about scaling to Nigeria with the same partners as in Greece and Somalia, the project was brought to a halt by the organization’s top management. The data scientist explained to us, “*The challenges with projects like [Carter], where you are free to select whatever you want, is that there is no framework. There are no predecessors that can guide you.”* Even more importantly, there was no AI or other assessment framework at the time for guiding the global-political use of the application. The project manager went on to explain that there were issues with the perceived ethics of the tool because of the nature of the humanitarian work of the organization, and the biases that could be present in predictive tools and their data: “*If the predictive tools do not work correctly, we are risking human lives, not responding well, or prioritizing something over something else which is a very serious decision with big consequences*.” The data scientist further explained that the organizational culture in the country offices was risk averse and not ready for such a tool: “*I remember a country information management officer in Somalia telling me, ‘I don’t think Org C is ready for [Carter]. Wait for 5 years, maybe in 5 years they will be, but not right now*.’”

The above concern resulted in the *Orchestrator*working with an academic institution to create a spin-off of the project through the inclusion of satellite imagery for understanding the interrelation between weather/climate anomalies, conflict and displacement. This highlighted the layered modular nature of the tool whereby its generativity made it possible to source new partners and to expand on the tool’s use and to create further functionalities. This collaboration continues to be expanded on by the group joining forces with a global group of volunteer AI engineers that work to solve specific challenges. The team explained, “*There is a spin-off. And they are trying to expand the scope to work more regionally*.” By September 2020, the spin-off was still in its development phase.

### Desai

#### The challenge

Org D is the UN agency aimed at improving reproductive and maternal health worldwide. In 2017, the Afghanistan country office reached out to HQ, the *Orchestrator*, to ask for help mapping and completing the country census. The last census in Afghanistan was done in 1978, which meant that the government and development agencies did not have a complete picture of important variables for decision making. Countries like Afghanistan were lacking basic data to understand where to build hospitals or new schools or how diseases, vaccines, and conflict impacted on their populations. This wicked problem is difficult to answer as it requires open knowledge and data flows that are obtainable from multiple, highly varied locations and organizations.

#### Solution development

As explained by the *Orchestrator* technical lead, at the beginning, the initiative sought to tackle the census problem in Afghanistan:

[Afghanistan] had 12 provinces out of 34 where they connected data, but the other provinces were just inaccessible or insecure or there was too much mobility going on so that they couldn’t do a full census in the entire territory. They asked if we had an idea of what could be done in the absence of official stats. We had just come to know [a research program at a UK university] and [an international NGO], together we called the Afghanistan country office who spearheaded the modeling population aspect.

The query from Afghanistan led to an experiment to understand how to use those data that were collected in the 12 provinces, and to use that to create correlations and interpolations by combining the limited data available with the satellite imagery from provinces where no data could be collected from. The technical lead explained how the *Orchestrator* started to experiment with the country office (the *Integrator*) and various data sources to get the tool off the ground. “*We used high resolution satellite imagery that already existed…used [a research partner] to develop the data sets further using survey data…funded our own micro-census to get sample data on which we can then further model*.” This highlights the recombination potential to create new value added, given the layered, modular nature of technology plus the data.

#### Ecosystem: Location 1

After a year and a half of production and many challenges, the team was able to provide solid results to the UN leadership and to the Afghanistan government. “*The President was really happy; the UN was really happy and finally we had numbers at the district level.”* The initiative was officially launched in January 2018 as *Desai,* comprising a geo-referenced infrastructure that provided the demographic and other data usable for development purposes. The geo-spatial layer and built-in multifunctionality creates the possibility for many applications to be developed from the initial layer, highlighting the generativity of the tool. In this way, *Desai* combined the expertise of *Complementors* in government, UN, academia, and the private sector to design adaptable and relevant geospatial solutions. The scaling process and importance of the *Integrator’s* role was explained by the initiative lead:

In a first step, country offices ask us for the tool, then we go there and understand the problems they are dealing with and the needs on the ground. I was in South Sudan in February and it’s hard to imagine how difficult the situation is on the ground. The government is unable to get the data and innovative solutions are necessary or it would take years.

Org D country offices are allocated a geospatial mapping expert to help implement the project as well as training on geospatial mapping, as explained by the initiative lead:

To increase the capacity in the country office, we develop trainings in open source or the tech of choice that would enable our government partners and local country staff to be able to translate the data into real decision-making power. They can answer questions such as where to develop future sites for schools, for hospitals.

#### Ecosystem: Location 2

Eventually, as the tool began to scale to new countries such as Nigeria in 2018, different *Integrators* started to join the project. For example, a global foundation came on board, enabling the channeling of funding from a large government (which had provided a grant for the project), as well as to aid with the implementation of an initiative for polio vaccine mapping. The foundation was looking to measure population growth and create more effective vaccination campaigns, particularly in Nigeria. An academic institution was brought in as an extra integrator to work on the foundation’s interests, but this led to a couple of challenges*,* in particular, *“in determining roles and responsibilities of the partners in the project.”* The local country office of Org D in Nigeria was also involved as an *Integrator*, along with other partners needed to raise funding and to connect to end-users. The Nigeria application was intended for assessing the effectiveness of the polio vaccination campaign using the census data.

#### Ecosystem: Location 3

The project was next scaled to the Democratic Republic of the Congo (DRC) in 2019. There, the effort focused on adapting the application to use census data to support a six-month rapid response measles vaccination campaign. The effort was intended to end an epidemic that had killed more than 6,000 people that year. New health-related government *Complementors* came on board in the country for the broader application of the technology. The *Desai* project team created the key data layers needed to produce the maps for targeting the interventions. These layers included settlement names and locations; settlement extents; health facilities; health zones and health area boundaries; and road data. In addition, the technology was built up in DRC to add in a semi-automated approach for creating maps.

What became challenging for the *Orchestrator* was the implementation issues involving scaling across different countries. The main issue as explained by the initiative lead was the differences in working speed between the various organizations, particularly with strict timelines and country engagements, which she said, *“…was not in line with how UN agencies operate.”* The recruitment of new staff in the HQ was also slow, and lead to difficulties in keeping up with demands of partners. Eventually the presence of the country offices on the ground as *Integrators* enabled Org D to get their footing back: *“There are no doubts about responsibilities anymore.”* By September 2020, the tool had been scaled to Burkina Faso, DRC, Ethiopia, Ghana, Mozambique, Namibia, Nigeria, Sierra Leone, South Sudan, and Zambia.

## SUMMARY OF FINDINGS

**Figure 1:**
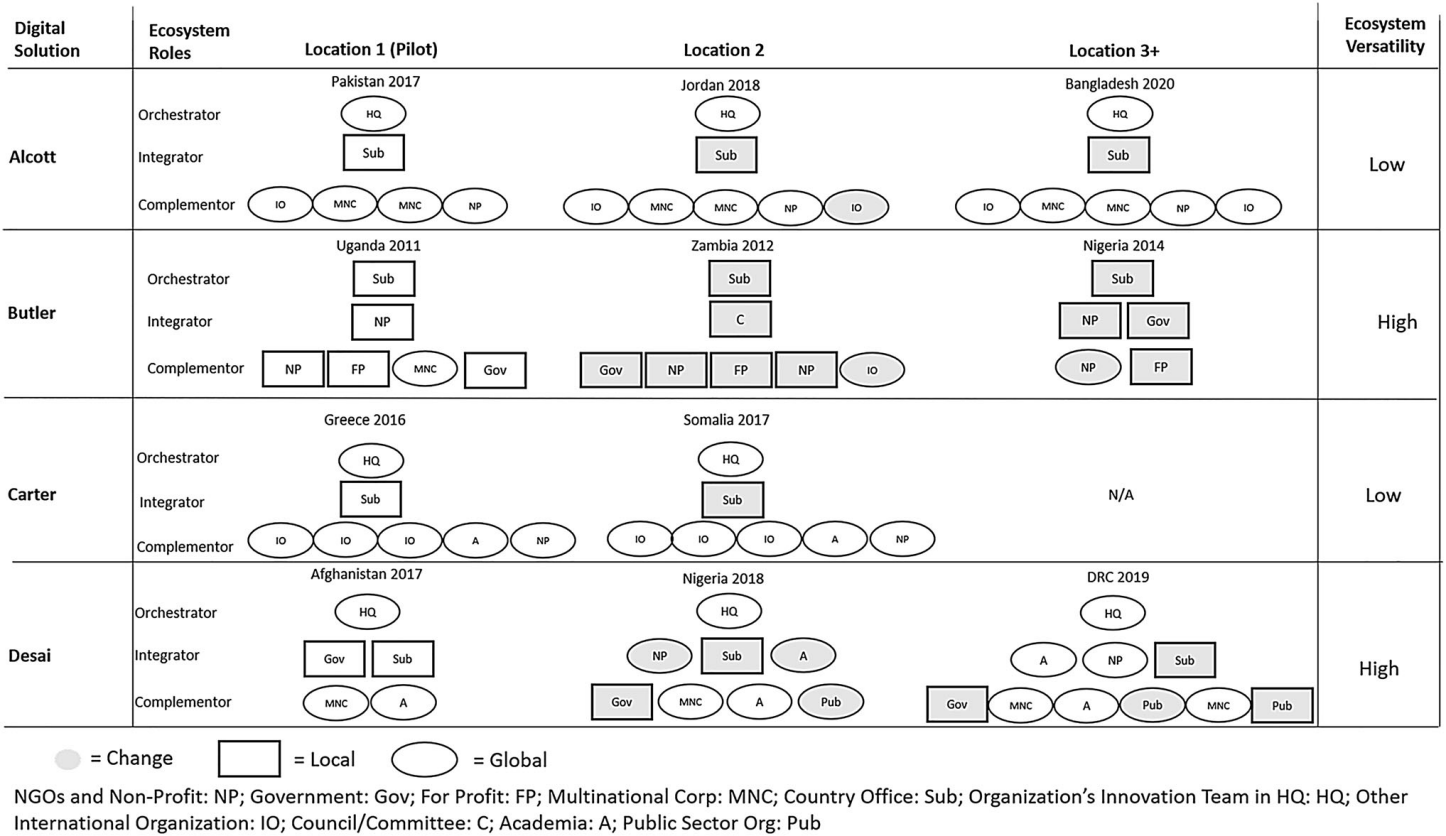
Evolution of ecosystem configurations during scaling.

Our findings describe how the ecosystems around digital solutions led by UN organizations were configured and how they changed during scaling. Cross-case analyses resulted in a detailed mapping and comparison of the ecosystem actors in each new location and provided deep insights into their roles. Figure 1 and Appendix I–IV offer an overview of these inductively deduced roles and the activities these actors performed as summarized in the rich text above. As we can see in these detailed representations, there are some clear differences in how the four ecosystems developed as they scaled. These results of our cross-case analysis will guide the discussion in the theory development.

## THEORY DEVELOPMENT

### Ecosystem Roles for the Scaling of Digital Solutions

As portrayed in the findings section, the categorization of roles as orchestrator, integrator, and complementor fittingly describes the configuration of the ecosystems. Our analyses further revealed the value their activities created for the international scaling of digital solutions. Within the broader strategy and innovation literature, the orchestrator role is played by a focal firm such as the HQ or subsidiary with the main activity of driving and setting up the ecosystem around the value proposition and focal firm’s strategic intent (Dhanaraj & Parkhe, 2006; Iansiti & Levien, 2004). Beyond the hierarchically determined phase of blueprint creation and partner role-orchestration typically seen in the strategy literature (Autio & Thomas, 2020), in our cases the orchestration of the ecosystem during the scaling phases takes on the activities of legitimization, network activation, and organizational responsibility, suggesting that ecosystem orchestration could be viewed as a form of dynamic capability (Teece, 2007, 2014). Like in prior studies (Nambisan et al., 2019), the orchestration capability facilitated both internal (intra-actor) and external coherence. Our cases highlight the importance of the orchestrator in aligning parties in support of an idea and its eventual program of work. In this sense, the orchestrator must manage both the problem framing and solution (from the outset) – two aspects that then involve the roles of complementor and integrator (Brusoni & Prencipe, 2013).

Another important role in the ecosystem is the complementor. Aligning with Williamson and De Meyer (2012), our findings show that complementors include several functional roles that provide value creating activities such as technology development, funding, market access, research, visibility, and delivery. These complementary services and functions aim to extend the application. In our cases, a variety of organizations, and not only those related to technology (Oborn et al., 2019), step into this role. In Alcott and Carter, the complementors were global actors which stayed in relatively stable positions during the ecosystem’s evolution. But with *Butler* and *Desai*, the complementors changed between local and global partners, depending on the end-user needs in that location. Developing these new partnerships was crucial for the ecosystem to activate the potential affordances of scaling the digital solution as it engendered a recombination of knowledge for new uses. In general, our cases support the insights that the success of digital solutions depends on scale, reconfiguration and continuing iterative advances (Ansari et al., 2016; Nambisan et al., 2017).

Most importantly, our findings shed new light on the nature of the integrator, which connects the orchestrator and the complementors, and channels information between them by providing access to local knowledge and partners. Prior studies have not separated the integrator role from the orchestrator (Dedehayir et al., 2018), but our findings show that in the context of the international scaling of digital solutions, the layered and modular nature of the technology further amplifies the integrator role, as it enables new integrators to participate in new locations (this is especially the case with *Desai*). This is key to enabling the technology’s generativity, and the creation of information or knowledge feedback loops, through facilitating the participation of diverse end-users, each with specific needs. At the same time, the changes in integrator- and complementor-partners reflect the shifting context of the highly contextual knowledge used in such applications. The integrator’s role builds on its knowledge of the potential partners on the ground, as well as the global perspective it provides to address these local problems.

The integrator role may be particularly salient in the focal (non-profit) context, where the orchestrator has less control and fewer resources for actively configuring local ecosystems in dispersed locations. We also suggest that technology projects for complex problems generally require not only technical implementation issues and the corresponding project management, but additional roles that “integrate” the soft knowledge of requirements, stakeholders’ needs, buy-in and actions, and partner incentivization (where financial motives are not strong enough). In summary, our results specify different ecosystem roles, and their activities as critical for the scaling of digital solutions. We show these in Table 3 and suggest the following proposition for future research:

#### Proposition 1a:

 The Orchestrator mobilizes the µü scaling of the digital solution.

#### Proposition 1b:

The Integrator enables access to local knowledge and partners – activating the affordances involved in the scaling of the digital solution.

#### Proposition 1c:

The Complementor provides value adding services and resources to implement the solution in the new location and grants access to the end-user – enabling the generativity necessary for the digital solutions to scale.

**Table 3:** Ecosystem roles for scaling of digital solutions

Ecosystem roles	Primary activity	Driver of ecosystem configuration in international scaling
Orchestrator	Ideate and set up the ecosystem around the idea (Dhanaraj & Parkhe, 2006; Iansiti & Levien, 2004)	Legitimization Network activation
Integrator	Contextualize and provide local knowledge	Knowledge access Partner access *Enables the affordances and recombination of knowledge to create new application avenues
Complementor	Assist in on the ground delivery and access to end-user through value-creating activities (expanding on Williamson & De Meyer, 2012)	Delivery Technology development Funding Market access Research Visibility *Enables access to end-user for generativity and data

### How Digital Solutions Enable Replication and Adaptation

The cases reveal that the international scaling of digital solutions requires complex interactions of the core digital technology and the ecosystem. While the core technology remained stable throughout the international scaling (for example, the blockchain itself never changed), our cross-case analysis revealed that the cases differed in the local application of the solution and the ecosystem participants. What we noted most importantly was that the three properties of digital solutions (modularity, generativity and affordances) impacted the adaptation and replication of the way the tool was applied in each new location.

While each solution exhibited all three characteristics, we have clear examples of certain properties of the digital solutions linking to the replication versus adaptation decision. For example, when we consider replication: *Alcott* was designed for a particular functional purpose – transferring money via the blockchain – and its application did not vary significantly across countries. The driving element of this replication was the modular property of the technology that allowed new technology modules to be plugged into the original tool. As we saw, different identification mechanisms (iris scanning or QR codes) could be plugged into the base technology keeping the application largely the same, even with the new elements. Similarly, despite being applied to different uses, *Desai* pursued a rather standardized application approach being essentially a geospatial tool with the potential to add on layers or build functionality depending on the local needs.

When it comes to adaptation, *Carter* was adapted by moving from the use of predictive analytics by applying meteorological data to population movements, to the use of predictive analytics based on new local variables for measuring population displacement from Somalia. This was enabled by the reusable nature of the tool, as new data sources changed with the functions embodied in the solution. *Butler* also involved different applications (depending on the needs of the country, its population, and its priorities) that were situated to specific cultural settings and knowledge. Most interesting is that, as the solution scaled, contextualizing its goals and usage to each location, its technological capabilities also developed over time – moving from being a pure SMS platform to using social media platforms, digital messaging providers, and eventually AI enabled chatbots. This illustrated the affordances of the tool as it was co-created with end-users and local working groups.

These observations lead us to present the following proposition:

#### Proposition 2a:

When scaling internationally, digital solutions’ modularity enables replication across locations.

#### Proposition 2b:

When scaling internationally, digital solutions’ generativity and affordances enable adaptation across locations.

Our theorizing on how the properties of digital solutions relate to replication and adaptation provides a new perspective on internalization theory. Traditionally, replication and adaptation are seen as a trade-off. But our findings show that they are differently-enabled by digital properties and recombinative characteristics. This is aggravated by the wicked nature of problems in the developing regions, which embody the added challenge of ‘rugged landscapes’ (i.e., challenging business environments) (Foster & Heeks, 2013; Kumar, Nim, & Agarwal, 2020; Oborn et al., 2019). The IB literature suggests that the mechanism consists of the *“recombination or bundling of existing FSAs (firm-specific assets) with country-specific advantages (CSAs) available in a host country”* (Li et al., 2019: 1449)*.* However, our focus on the properties of digital solutions reveals that replication and adaptation are enabled by both the generativity of the core technology allowing its reuse across borders, as well as its inherent modularity and affordances. The latter allows the ecosystem partners to develop and enhance solutions according to local needs (as seen in all four cases). This shows that the replication-adaptation trade-off may be overcome by the properties of digital solutions, allowing for a simultaneous replication and adaptation.

### Ecosystem Versatility for Digital Solutions

In addition to the impact of the digital properties on replication and adaptation, we found that the international scaling of digital solutions often involves ecosystems being configured anew in each location. This requires us to think differently about local ecosystems and partners, and to move away from the lead firm perspective typically adopted in innovation ecosystems research. Instead, our findings highlight the value of local cultural knowledge and domain knowledge, as well as users’ co-creation of innovations (Busch & Barkema, 2021; Foster & Heeks, 2013). They support recent research showing that businesses using a core application were found to “generate” additional services integrated with the core but also to create extensible applications on a ‘topmost’ layer, which allows it to scale across more users (Oborn et al., 2019). To that, we also noted that the modular, layered nature of technology can also bring in new knowledge from new users and partners. This refines the role of the integrator, making it separable from the orchestrator, while also making a variety of complementors possible – this all due to the digital nature of the technology. We can also infer that while these roles are involved in recombining for new local ecosystems, they must also cover the need for different kinds of resources – both local and global – operating at different layers – technology, content and otherwise.

The adaptation to a new context may then involve new kinds of creative interactions with local users, resulting in a high level of Ecosystem Versatility, that is: changes in the ecosystem configuration across locations. These changes can concern both the type of actor involved as well as their global-local reach. Our findings on the reconfiguration of ecosystems may be particularly salient in the UN context, but generally highlight the need for ecosystem versatility in development contexts which are often characterized by a lack of formal institutions, political risk and corruption (Sartor & Beamish, 2014; Sun et al., 2021; Vaaler, 2008). In such a context, configurations must be tailored to the availability of capable partners (Stadtler, 2018), resulting in a need for versatile ecosystems. These must be facilitated by the Orchestrator (usually the organization’s HQ) with cross-sector partners as Integrators that have a global outlook with a deep local grounding. This points once again to the critical role of the Integrator for digital solutions. Our cases showed a high level of ecosystem versatility for *Butler* and *Desai* (see Figure 1). These two cases had different configurations of Integrators for responding to the complex environments of those countries, and where the country office did not have the capacity to fulfill the Integrator role. Thus, we propose:

#### Proposition 3a:

 Ecosystem Versatility is higher when Orchestrator–Integrator relationships are cross-sectoral and/or inter-organizational.

*Alcott* and *Carter*, however, it was primarily the complementors that were reconfigured across locations while the Orchestrator–Integrator relationship was always an intra-organizational HQ–subsidiary dyad (as we would expect in an MNC). Consequently, we propose that strong HQ-subsidiary relationships will facilitate a higher level of ecosystem replication across locations and thus limit the need for versatility in development contexts.

#### Proposition 3b:

Proposition 3b: Ecosystem Versatility is lower when Orchestrator-Integrator relationships are hierarchical intra-organizational.

In contrast to many previous IB studies, ecosystem versatility as a concept goes beyond a perspective of there being a replication or adaptation across countries. Ecosystem versatility is associated with the diversity of stakeholder configurations found in the development context (rather than just country differences) and exposes the global and local locus of organizations for scaling digital solutions. It also captures intra-organizational as well as inter-organizational relationships allowing us to see all dynamics. These insights provide a further extension to recent work on the conceptualization of ecosystems (Parente et al., 2019; Li et al., 2019), specifically for international scaling.

### A Typology of International Scaling for Digital Solutions

Based on the theoretical dimensions we identified above, we derive a typology to specify which property of the digital solution– either modularity or generativity/affordances – is driving the scaling; and which level of ecosystem configuration – low versus high – is present. This results in four types of international scaling of digital solutions at the intersection of “application adaptation” and “ecosystem versatility”.

**Figure 2:**
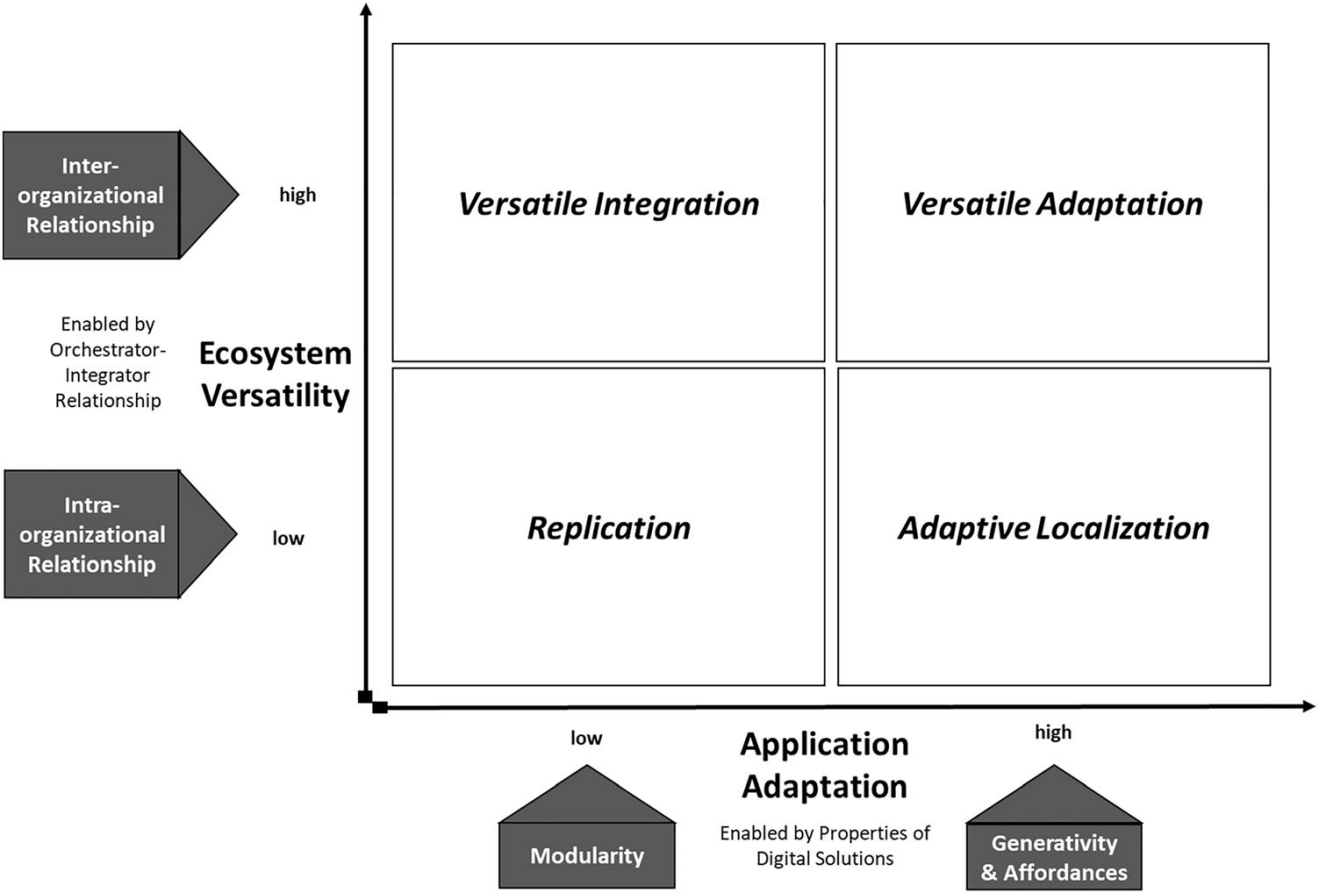
Theoretical framework: international scaling types.

Application adaptation refers to the extent to which the application of the tool was adjusted to the specific requirements of the new location and the different levels of ecosystem versatility capture the change in the ecosystem across locations. The two dimensions provide useful distinctions of different scaling types. By illustrating this level of granularity on the different components of a scaling digital solution, we provide a new view on the traditional IB replication-adaptation dilemma (Jonsson & Foss, 2011; Szulanski & Jensen, 2006; Venaik et al., 2004). The concept of ecosystem versatility relies on the intra-organizational and inter-organizational relationships usually highlighted in the IB literature, except that in the ecosystem perspective, these relationships are manifested through co-creation efforts and role fluidity (Boley & Chang, 2007). Based on this emergent theoretical understanding, we suggest that the interaction of ecosystem versatility and application adaptation result in four different types of international scaling for digital solutions*.* As shown in Figure 2, these four types address the replication-adaptation dilemma in different ways. Subsequently we mapped the four cases on this typology.

The case of *Alcott* shows that modularity plays an important role in the scaling process as it moves from refugee camp to refugee camp, where interfaces in the core technology enable the ease of integrating existing technologies in those locations. Also, the ecosystem’s configuration remains very stable across locations. The Orchestrator–Integrator dyad refers to the HQ and the field offices (subsidiaries) that collaborated with similar types of local partners. These patterns align more closely with the IB literature on the *replication* of products (Jonsson & Foss, 2011). *Carter* portrays the second way of how a digital solution can scale: by maintaining the ecosystem configuration with a HQ-subsidiary pattern while changing the local application of the tool significantly across locations. The generative nature of the tool allowed for the introduction of new data sources to create new functionalities. We call this *adaptive localization*. The third type of scaling is through replicating the application but adapting the ecosystem with each new location. We found that in this case the challenges of the development context cause a massive reconfiguration of the ecosystem in each new location, resulting in high levels of ecosystem versatility. For *Desai,* close interaction with the local public sector was key for the success of its solution and amplified the affordances based on the nature of the tool and institutional end-user where partners had to be adapted in each new country. We call this *versatile integration*. And finally, the fourth way is for the digital solution to change not only in its ecosystem but also in the application of the solution. This *versatile adaptation* was seen in the *Butler* case, which adapted its partners in each new location as well as by changing the problem focus of the tool, despite continuing to use the same core technology as the system’s backbone.

## DISCUSSION

Addressing the question of how digital solutions for wicked problems are scaled across locations in development contexts, our study makes two contributions to the literature: on the ecosystem’s roles, and on the prominent IB dilemma of replication-adaptation.

First, we uncover different ecosystem roles and their contribution to the international scaling of digital solutions. Like Nambisan et al. (2019), we find that digital solutions not only radically reshape the nature and structure of the global economy but also the interconnected nature with which MNCs can work with organizations in other industries to address the world’s most pressing needs and identify the specific roles necessary for such collaborations. These insights add to the scarce literature that has focused on specifying the roles of different ecosystem actors (Williamson & De Meyer, 2012) and the roles played in digital solutions ecosystems for global reach (Li et al., 2019; Nambisan et al., 2019).

In the context of the UN, the use of the orchestrator-integrator-complementor framework not only revealed the deep embeddedness of local problems but also the need for digital solutions to be managed by networks of different partners (rather than by being owned by a single organization), as shown in previous work on distributed social innovations (Oborn et al., 2019). Due to our specific research context, we uncover the role of the orchestrating organizations (the UN agencies) as ethical gatekeepers in digital solution scaling. We see that the UN agencies were able to add value through their global charter and status, as well as through legitimacy created over many years of access to local partners and beneficiaries globally. This provides an interesting avenue for future researchers looking to understand business ecosystems, particularly as digital solutions are facing increasing ethical concerns, both in the non-for-profit as well as in the for-profit domain. Orchestrators may also serve the ethical role but need to interact with and be informed by their complementors, as technologies can change, and applications’ consequences can be unintended, or only realized after initial experiments. The rise of tech savvy “digital natives” across populations is leading to important transformations, including global societal changes (Vodanovich, Sundaram, & Myers, 2010) and complementors can act as the link to enable a change in the interaction between the organizations and these user-beneficiaries. The access to data is also transformative for many organizations (Davenport et al., 2012) to understand their beneficiaries and their needs. Our study showed the critical role of the integrator, a role previously not well defined in the literature, possibly due to its incorporation into the focal firm as orchestrator. Given the layered nature of digital technology, in a cooperative setting, the orchestrator can afford to give up its technical architecting responsibility, and to focus on soft issues. We see that often large bureaucratic organizations with limited technical capabilities are unable to connect with the complementors directly (Ambos & Tatarinov, 2022) and require partners to source localized knowledge. While the emphasis on the integrator role may be context-bound, we propose that the three different roles are generic and driven by the characteristics of digital solutions.

Second, our findings show that combining properties of the digital solution with the ecosystem configuration is key to understanding how digital solutions scale internationally*.* The four types of scaling – which vary in their ecosystem versatility and local application adaptation – reflect the insights of this research for the extant literature on internationalization and the replication-adaptation dilemma. Our findings suggest that this trade-off may be overcome as digital solutions allow simultaneous replication (through their modularity) and adaptation (through their generativity and affordances). Because these properties are always interconnected, they enable the digital solution to scale across borders faster and more seamlessly than traditional technology products (Nambisan et al., 2017). This also highlights the need for ecosystem versatility, which puts a significant burden on the orchestrator organization driving the scaling. Versatility is a necessity in development contexts where many stakeholders must be involved and shows the need for the localization and adaptation of digital solutions through different partner configurations, to create greater impact and value (Busch & Barkema, 2021; Chliova & Ringov, 2007; Kistruck et al., 2013). In short, our framework provides more granularity on the theoretical dimensions of the replication-adaptation dilemma. While the IB literature has traditionally focused on product features that are replicated or adapted, when analyzing digital solutions, combining the properties of the digital solution with the ecosystem configuration characteristics is key to understanding how they scale.

## LIMITATIONS AND CONCLUSION

This research sheds light on the challenges of scaling digital solutions that seek to address wicked problems. Applying an inductive research design, we provide fine-grained detail on the global-local and intra- and inter-organizational dynamics of ecosystems during the international scaling process. The limitations of this work are related primarily to its inductive nature and its specific context. While taking great care to avoid informant biases and retrospective sense-making through the methodology, the nature of the data does not allow us to rule out biases completely. The variety of fields and applications in the study also made it difficult to develop an even finer-grained measurement of the impacts of the partnerships within the ecosystems, which can be an important avenue for future studies. The specific narrow context of wicked problems can also act as a limitation as it remains to be seen if our findings would be generalizable to for-profit oriented solutions. Another limitation of the study is that due to the challenge of identifying rare cases and gaining detailed data access, the solutions were sampled from four different organizations and based on different types of technologies. While the data showed neither systematic variation across these organizations nor were related to the type of technology employed, we recommend future research in one single organization to create more detailed accounts on specific organizational processes and contingencies. We would also envision future research to test our propositions and collect quantitative data around the new constructs we develop.

The choice of our research context was based on the salience of the focal phenomenon. Naturally, our findings are embedded in this specific context of digital solutions driven by UN agencies and their relevance to a pure for-profit context will have to be confirmed by future studies. However, it may at least inform MNCs how to drive greater cross-industry collaboration when addressing the world’s wicked problems, with the additional ecosystem parameters needed to fully embrace ‘base of the pyramid’ business conditions. Effectively, through the fine-grained ecosystem maps that emerge in our findings, we build deeper knowledge on a previously overlooked phenomenon in IB and help to elevate new perspectives on the traditional replication-adaptation dilemma by including digital technology properties and ecosystems. We urge future researchers to continue moving in this direction of bridging the social-business divide and to explore the coordination mechanisms needed in ecosystems for global scaling of digital solutions.

## Notes

1. Names have been anonymized.
